# Toads are plastic, it’s fantastic! Or is it?

**DOI:** 10.1093/conphys/coy048

**Published:** 2018-09-05

**Authors:** Kim Birnie-Gauvin

**Affiliations:** Section for Freshwater Fisheries and Ecology, National Institute of Aquatic Resources, Technical University of Denmark, Vejlsøvej 39, 8600 Silkeborg, Denmark

We’ve all heard about the infamous cane toads introduced in Australia in an attempt to control the beetles that were decimating sugar cane crops. In June 1935, the decision to introduce these feral beasts was put into action as a means of replacing harmful pesticides. But, the situation went from bad to worse at (almost) the speed of light. Cane toads now number over 200 million (!!), they contribute to the spread of diseases, have reduced biodiversity, but *have not* affected the cane beetles they were meant to eat. Oops!

So what makes these invaders so successful that they have already invaded more than 40 countries? The answer lies—at least partly—in the cane toads’ plastic nature, their ability to adapt to changing environments. The cane toads were initially introduced in areas of Australia where the climate was tropical, and similar to that of their native range. However, the toads rapidly moved West and South, reaching areas where the climate was colder and very different from that of their native range. Remarkably, within a few hours of exposure to colder conditions, the toads were able to adjust their cold tolerance (i.e. the lowest temperature they can withstand).

McCann and colleagues wondered whether this ability to regulate lower thermal tolerance in response to cold temperatures was only present in those individuals that had conquered the colder fronts, or whether it was present in all cane toads. The team studied almost 300 cane toads from warm and cold regions of Australia (the invaded country) and Hawaii (the origin country). They first acclimated them to either cold (12°C) or warm (24°C) temperatures for 12 h. Then, the researchers lowered the toads’ body temperatures slowly to see how cold they could get before they could not right themselves if turned on their backs. This temperature would be known as their critical thermal minimum.

And as you probably predicted, it turns out that the toads that invaded the cold regions actually have a lower critical thermal minimum than those from warm regions.

At this point, it remains unknown whether this ability is genetically-driven or develops with exposure. However, if it is coded genetically, the cold conquerors could pass on their ability to their offspring, which may make cane toads even more successful in the future. There is no doubt that the cane toads’ ability to acclimate and thrive in highly variable conditions has increased their invasion success beyond what was initially predicted. In fact, there are no other reports of such dramatic changes in an organism’s critical thermal minimum occurring in such a short timeframe (<80 years) in any other species. Cane toads truly are plastic, but that’s not so fantastic.

**Figure:**
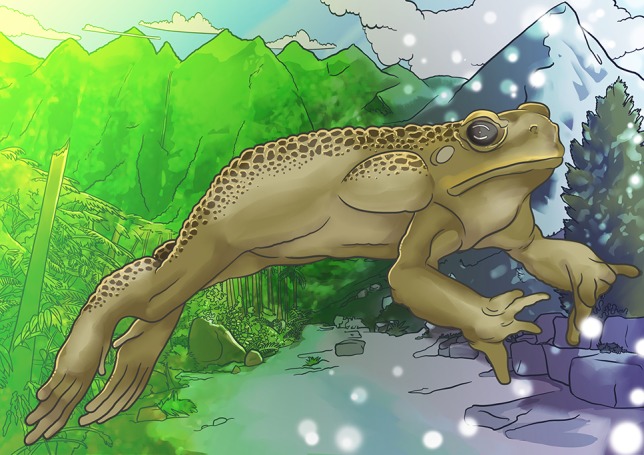


Illustration by Erin Walsh; Email: ewalsh.sci@gmail.com
